# The Corning Typhoid Epidemic

**Published:** 1912-06

**Authors:** 


### The Corning Typhoid Epidemic

THROUGH the courtesy of Dr. Frank S. Swain, Health Officer of Corning, we are able to present a timely report on the epidemic in progress in that city. Bearing in mind how busy he is at this time, with both private and public duties of an urgent nature, our readers will appreciate this courtesy the more.

The comparative mildness of the epidemic in connection with the fact that there was a precursory epidemic immediately following the contamination of the water, may have great significance in regard to the mutual relations of colon and typhoid bacilli, especially as to a protective influence of one infection upon another.

What we wish to emphasize most, however, is the hopefulness with which such problems can now be dealt as contrasted with the time, scarcely a generation distant, when such an epidemic was still regarded as a visitation of Providence by a large share of the community and when the most learned and expert were powerless to prevent further progress or even to draw lessons for the future.

In a sense, such an epidemic is more truly a visitation of Providence now than formerly. It is a logical and, in the long run, inevitable consequence of a risk which, in the present case can apparently be definitely traced. The daily press of Corning which gives loyal and intelligent support to the work of relief and prophylaxis, goes so far as to ascribe the epidemic directly to the fact that the closure of a valve was neglected in what is normally the outlet of the reservoir but which became an inlet during the stage of high water in the river. In these days, every community knows or can know exactly what to do to prevent typhoid. The practical problem differs for different communities; there are more or less perfect solutions, often involving great differences in expense which must be considered. But, must each community delay the solution of the problem till its own punishment for neglect comes upon it?
own punishment for neglect comes upon it? The following press dispatch is a partial answer:

The city of Hornell has been directed by Dr. Eugene H. Porter, state commissioner of health, to show cause before him on June 20th, why action should not be directed against that municipality for polluting the Canisteo river through its system of sewage.

Complaint was also made against the cities of Elmira and Corning, but they have agreed to install new sewage disposal plants.

